# Thyroid-like low-grade nasopharyngeal papillary adenocarcinoma with squamous differentiation in the posterior nasal septum: A rare case report

**DOI:** 10.1097/MD.0000000000028349

**Published:** 2021-12-23

**Authors:** Li Yi, Honglei Liu

**Affiliations:** a Department of Pathology, Second Hospital of Hebei Medical University, Shijiazhuang, China; b Department of Neurosurgery, Shijiazhuang Third Hospital, Shijiazhuang, China.

**Keywords:** nasopharynx, posterior nasal septum, squamous differentiation, TL-LGNPPA

## Abstract

**Rationale:**

Thyroid-like low-grade nasopharyngeal papillary adenocarcinoma (TL-LGNPPA) is a rare neoplasm characterized by papillary epithelial proliferation and abnormal expression of thyroid transcription factor-1. To our knowledge, squamous differentiation in TL-LGNPPA is an unusual phenomenon, and only 1 case has been reported to date. The tumor occurs mainly on the roof of the nasopharynx. Herein, we report a case of TL-LGNPPA with squamous differentiation located on the posterior nasal septum.

**Patient concerns:**

A 45-year-old Chinese women presented to our hospital with a complaint of nasal obstruction for 10 years and the symptom has been getting worse for recent 3 years.

**Diagnoses:**

Microscopic examination of the tumor tissue revealed findings typical of TL-LGNPPA, and in addition to the typical components, squamous differentiation could be found in some areas within the fibrovascular cores of the papillary components. Immunohistochemically, the squamous cells were positive for P40 and P63 but negative for thyroid transcription factor-1.

**Interventions:**

The patient underwent complete resection of the tumor.

**Outcomes:**

Follow up results showed that the patient had no recurrence 41 months after removal of the tumor.

**Lessons:**

Our report anticipates that, although TL-LGNPPA with squamous differentiation in the posterior nasal septum is rare, this report will expand the existing knowledge associated with TL-LGNPPA.

## 1 Introduction

Epithelial tumors of the nasopharynx are classified as either nasopharyngeal carcinoma, nasopharyngeal papillary adenocarcinoma, or salivary gland-type carcinoma according to the 2017 World Health Organization Classification of Head and Neck Tumors.^[1]^ Thyroid-like low-grade nasopharyngeal papillary adenocarcinoma (TL-LGNPPA) is an extremely rare type of nasopharyngeal papillary adenocarcinoma. Nine cases were first described and characterized by Wenig et al^[2]^ who named LGNPPA in 1988. Carrizo and Luna^[3]^ described 2 pediatric cases, indicating that these tumors were similar to papillary thyroid carcinoma (PTC) for histological morphology; they documented the expression of thyroid transcription factor-1 (TTF-1) in tumor cell nuclei, and renamed them TL-LGNPPA. Because of the rarity of the neoplasm, TL-LGNPPA is still considered to be at the stage of case collection. Only 1 case of TL-LGNPPA exhibiting a squamous differentiation component has been reported previously in the English literature.^[4]^ This tumor is typically located in the nasopharynx arising from the roof or lateral wall of the nasopharynx, and in a few cases, it has been noted to be attached to the posterior nasal septum.^[5]^ Herein, we present another case of TL-LGNPPA with squamous differentiation located on the posterior nasal septum. Interestingly, the TL-LGNPPA exhibited a different histopathological finding in addition to different morphological and immunohistochemical features compared to previous cases.

## 2 Case report

**Figure 1:**
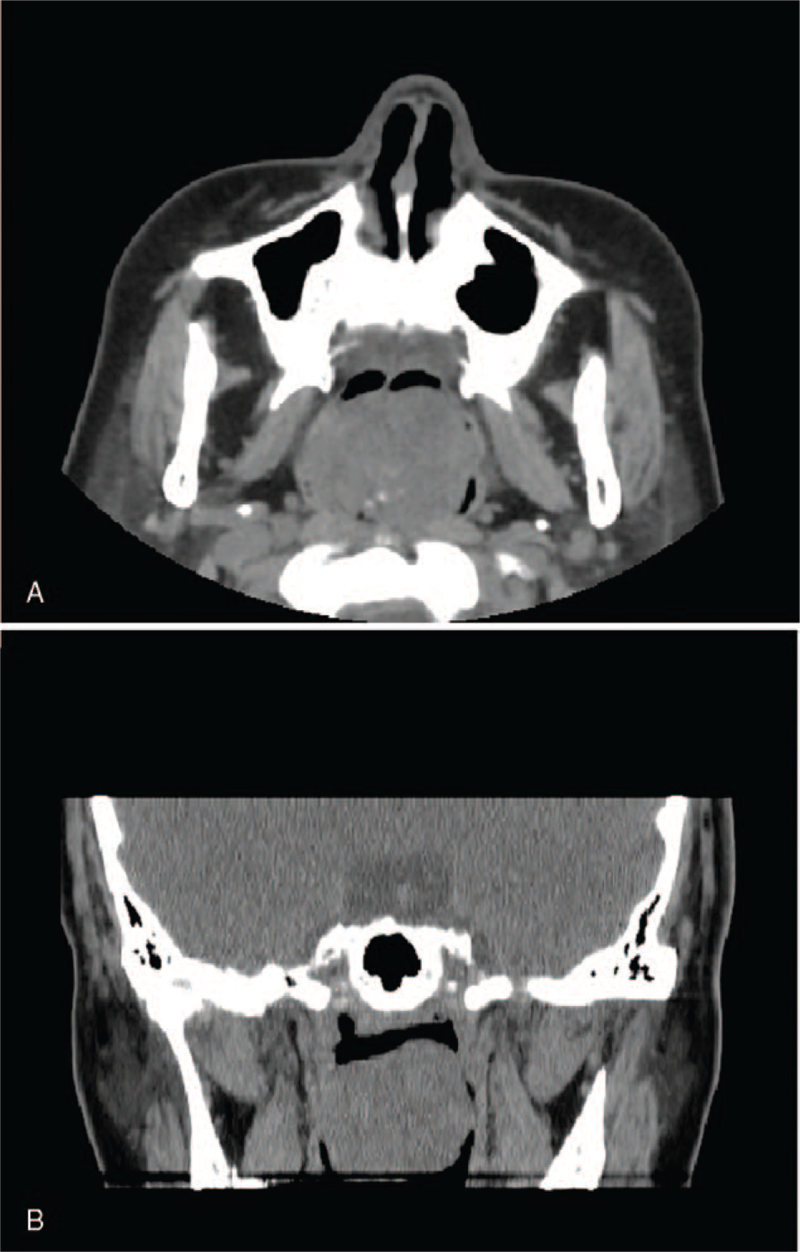
(A) Horizontal view image of head/neck CT scans shows a protruding mass in the nasopharynx (arrow). (B) A coronal view image of head/neck CT scans shows a protruding mass in the nasopharynx (arrow). CT = computed tomography.

A 45-year-old woman visited our hospital with a 10-year history of nasal obstruction that worsened over the past 3 years. On head and neck computed tomography, a horizontal view and a coronal view image exhibited a protruding mass on the posterior edge of the nasal septum (Fig. 1A and B). No swollen lymph nodes were observed. The mass was clinically diagnosed as nasopharyngeal carcinoma. Finally, the patient underwent complete resection of the tumor. Several days after surgery, the patient's nasal obstruction was relieved, and she was discharged from hospital. Three months later, computed tomography examination and pathological biopsy revealed no residual neoplastic tissue. The patient remained free of recurrence or metastasis after 41 months of follow-up.

**Figure 2:**
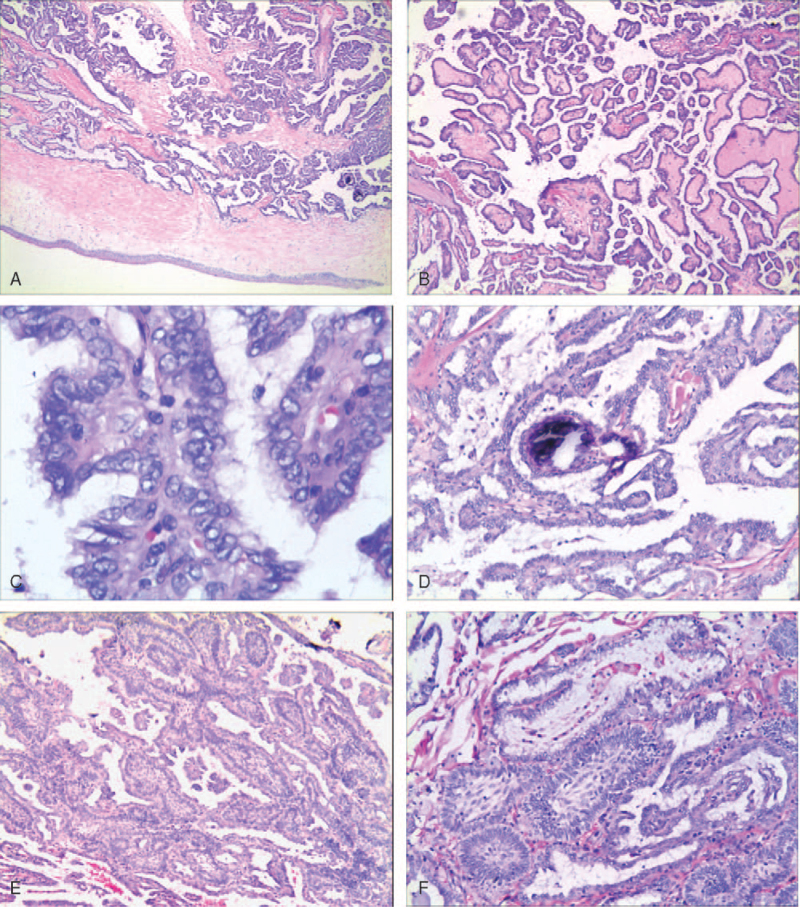
(A) The tumor growth under mucosa ×25. (B) The tumor was mainly composed of complex papillae with hyalinized fibrovascular cores ×40. (C) Nuclear overlapping, irregular thickened nuclear membranes, nuclear grooves, and ground-glass appearance were frequently seen ×400. (D) Scattered psammoma bodies appeared ×100. (E) Low-power view of the squamous cells gathered into clusters in the fibrovascular cores ×100. (F) The squamous cells showed no obvious atypia ×200.

Macroscopically, the tumor was described as an ash red polypoid with dimensions of 4 × 3.5 × 2 cm. Histopathological examination confirmed that the tumor was partially covered with normal respiratory epithelium and growth under the mucosa (Fig. 2A). The tumor was mainly composed of a papillary growth pattern; moreover, the fibrovascular core of the papillary showed hyalinization (Fig. 2B). Each papilla was covered by cuboidal or columnar epithelial cells with tiny bland, round to oval nuclei. In some areas, the nuclear appearance was very similar to that of PTC, including irregular thickened nuclear membranes, nuclear grooves, intranuclear cytoplasmic pseudo-inclusions, and ground-glass appearance (Fig. 2C). Necrosis was not observed, whereas scattered psammoma bodies were observed (Fig. 2D). In addition to the above common pathological features, the case showed an additional characteristic called squamous differentiation. Squamous cells were observed within some of the fibrovascular cores of the papillary configuration, packed closely, grown in a solid pattern with a lobular configuration, and prominent peripheral palisading; atypia and keratinization were not found (Fig. 2E and F).

**Figure 3:**
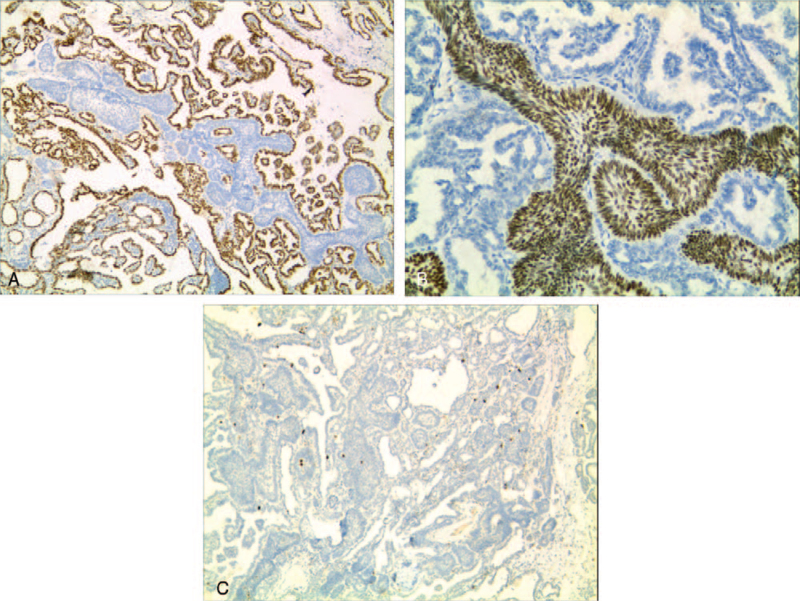
(A) TTF-1 expression was observed in the papillary tumor cells but negative in the squamous cells ×40. (B) The squamous cells show positive staining for P40 ×100. (C) The Ki-67 index was low in the area of papillary tumor cells and in the squamous cells ×40. TTF1 = thyroid transcription factor-1.

On immunohistochemistry, all tumor cells were positive for pan cytokeratin and epithelial membrane antigen, but negative for thyroglobulin. Additionally, TTF-1 exhibited strong nuclear immunoreactivity for glandular tumor cells but was negative for squamous cells (Fig. 3A). In addition, these squamous cells were positive for p63 and p40 (Fig. 3B). The Ki-67 index reached 3% in the area of the greatest concentration, and the squamous cells also showed low proliferating activity (Fig. 3C). We performed polymerase chain reaction to amplify exons 11 and 15 of 1v-Raf murine sarcoma viral oncogene homolog B1 ( BRAF ), and the results were negative.

## 3 Discussion

Most TL-LGNPPAs have a pedunculated and polypoid shape, and occur in any part of the nasopharynx, commonly observed on the top, lateral, and posterior walls of the nasopharynx as well as on the posterior edge of the nasal septum.^[5]^ The mean age of patients with TL-LGNPPA is 32.3 years (range: 9–68 years), and the incidence does not differ according to sex; the tumor size ranges from 0.3 to 4.0 cm.^[6]^ Because of its location, patients usually present with nasal obstruction and/or nasal discomfort, epistaxis, and headache. In this case, the neoplasm was located on the posterior edge of the nasal septum, which is a rare location. The other clinical manifestations were not distinct.

The presence of squamous differentiation in TL-LGNPPA is unusual. Only 1 case report describing TL-LGNPPA exhibiting squamous differentiation has been published to date.^[4]^ Histologically, the tumor showed the typical appearance of TL-LGNPPA. In addition, the presence of squamous differentiation is a major feature. The case described by Oide et al^[4]^ showed that scattered squamous cell foci were intermixed with glandular tumor cells. However, our case showed additional characteristics: the squamous cells were isolated within the fibrovascular cores and grew in a solid pattern with a lobular configuration and prominent peripheral palisading, but not intermixed with glandular tumor cells. Keratinization was not observed; however, the squamous cells showed no obvious nuclear atypia or proliferating activity in either of these 2 cases.

The immunohistochemical features of this case were consistent with those of previously reported cases of TL-LGNPPA: the glandular tumor cells exhibited diffuse strong immunoreactivity for TTF-1, but no immunoreactivity for thyroglobulin. The difference is that the squamous cells exhibited both p40 and TTF-1 immunoreactivity in Oide's case, but in our case, the squamous cells were positive for p63 and p40, and negative for TTF-1. BRAF mutations were negative in both cases, which is consistent with results reported previously in the literature.^[5,7]^

TL-LGNPPA with squamous differentiation is morphologically similar to the squamous metaplasia observed in PTC and needs to be differentiated from squamous cell carcinoma. Cytological atypia of the squamous epithelium is the key to differentiating carcinomas. In addition, squamous morules should be distinguished from squamous differentiation. Squamous morules are characterized by rounded aggregates or syncytial sheets of cells with bland round, ovoid, or spindle-shaped nuclei with indistinct cell borders; p63 is focally or diffusely positive in most of the squamous differentiation elements, whereas morules are usually negative.^[8]^ In the present case, the squamous cells showed no obvious nuclear atypia and were diffusely positive for p63 and p40; therefore, we believe that it is squamous differentiation.

The prognosis of TL-LGNPPA as a low-grade malignancy is excellent.^[7]^ Thus far, no case of TL-LGNPPA has been associated with lymphovascular spread or metastasis with follow-up periods of up to 15 years.^[9]^ Local recurrence may occur because of inadequate surgical resection. After resection of the tumor, the patient in our case was followed up for 41 months, with no recurrence. Follow-up information for the other case of TL-LGNPPA with squamous differentiation was unavailable for comparison. Some studies have found that squamous differentiation is associated with a poor prognosis of PTC.^[10,11]^ Because the number of cases reported is small, we do not know what this means for TL-LGNPPA with squamous differentiation. This suggests that we should follow up our patient for a long period of time.

The etiopathogenetic mechanisms responsible for the development of TL-LGNPPA remain to be elucidated. The majority of nasopharyngeal malignant tumors are squamous cell carcinomas and are associated with Epstein Barr virus (EBV). Therefore, many studies have used in situ hybridization and polymerase chain reaction to detect EBV in tumor cells of TL-LGNPPA,^[12,13]^ but no evidence of EBV infection has been found. Kakkar et al^[14]^ reported a case of TL-LGNPPA as a second malignancy five-and-a-half years after the patient was diagnosed and treated with radiotherapy for a diffuse astrocytoma in the frontal lobe. Therefore, they suggested that exposure to radiation could be a shared pathogenic factor. Considering the morphological similarity to PTC, some investigators have performed sequencing for mutations in BRAF and NRAS in cases of TL-LGNPPA, but the results were negative.^[15,16]^ Clearly the pathogenesis of TL-LGNPPA requires further research.

## 4 Conclusion

In summary, our case exhibited a rare histopathological finding in addition to morphological and immunohistochemical features typical of TL-LGNPPA with squamous differentiation. Although the posterior nasal septum has been involved in previous cases of TL-LGNPPA, this is the first case of TL-LGNPPA with squamous differentiation in this rare site. Hence, the report of this case extends the understanding of this tumor, in order to aid both clinicians and pathologists in making appropriate final diagnoses and increases awareness of this possibility.

## Author contributions

**Data curation:** Honglei Liu.

**Writing – original draft:** Li Yi, Honglei Liu.

**Writing – review & editing:** Li Yi.

## References

[1] El-Naggar AK Chan JKC Grandis JR. World Health Organization Classification of Head and Neck Tumours. 4th ed. Lyon, France: IARC; 2017.

[2] Wenig BM Hyams VJ Heffner DK. Nasopharyngeal papillary adenocarcinoma. A clinicopathologic study of a low-grade carcinoma. Am J Surg Pathol 1988;12:946–53. 2462370

[3] Carrizo F Luna MA. Thyroid transcription factor-1 expression in thyroid-like nasopharyngeal papillary adenocarcinoma: report of 2 cases. Ann Diagn Pathol 2005;9:189–92. 16084450 10.1016/j.anndiagpath.2005.04.019

[4] Oide T Kadosono O Matsushima J. Thyroid-like low-grade nasopharyngeal papillary adenocarcinoma with squamous differentiation: a novel histological finding. Hum Pathol 2017;70:43–8. 28601658 10.1016/j.humpath.2017.05.020

[5] Takakura H Hamashima T Tachino H. Clinicopathological features of thyroid-like low-grade nasopharyngeal papillary adenocarcinoma: a case report and review of the literature. Front Surg 2020;7:596796. 33330610 10.3389/fsurg.2020.596796 PMC7710863

[6] Mirza R Dela Cruz N Herrera GA. Thyroid-like low-grade nasopharyngeal papillary adenocarcinoma with biphasic histology. Case Rep Pathol 2020;2020:3275916. 32015924 10.1155/2020/3275916 PMC6988662

[7] Lee SH Kim H Kim MJ. Biphasic thyroid-like low-grade nasopharyngeal papillary adenocarcinoma with a prominent spindle cell component: a case report. Diagnostics (Basel) 2020;10:323. 32438756 10.3390/diagnostics10050323 PMC7277985

[8] Blanco LZ Heagley DE Lee JC. Immunohistochemical characterization of squamous differentiation and morular metaplasia in uterine endometrioid adenocarcinoma. Int J Gynecol Pathol 2013;32:283–92. 23518912 10.1097/PGP.0b013e31826129e1

[9] Petersson F Pang B Loke D. Biphasic low-grade nasopharyngeal papillary adenocarcinoma with a prominent spindle cell component: report of a case localized to the posterior nasal septum. Head Neck Pathol 2011;5:306–13. 21424532 10.1007/s12105-011-0252-4 PMC3173538

[10] Beninato T Kluijfhout WP Drake FT. Squamous differentiation in papillary thyroid carcinoma: a rare feature of aggressive disease. J Surg Res 2018;223:39–45. 29433884 10.1016/j.jss.2017.10.023

[11] Wan Sohaimi WF Lee YF Mat Nawi N. A case rarity: papillary thyroid carcinoma with squamous metaplasia complicated with chronic discharging ulcers. Indian J Surg Oncol 2019;10:676–8. 31857764 10.1007/s13193-019-00977-8 PMC6895325

[12] Li M Wei J Yao X. Clinicopathological features of low-grade thyroid-like nasopharyngeal papillary adenocarcinoma. Cancer Res Treat 2017;49:213–8. 27384157 10.4143/crt.2016.195 PMC5266392

[13] Ohe C Sakaida N Tadokoro C. Thyroid-like low-grade nasopharyngeal papillary adenocarcinoma: report of two cases. Pathol Int 2010;60:107–11. 20398195 10.1111/j.1440-1827.2009.02480.x

[14] Kakkar A Sakthivel P Mahajan S. Nasopharyngeal papillary adenocarcinoma as a second head and neck malignancy. Head Neck Pathol 2019;13:699–704. 29923095 10.1007/s12105-018-0944-0 PMC6854352

[15] Fu CH Chang KP Ueng SH. Primary thyroid-like papillary adenocarcinoma of the nasopharynx. Auris Nasus Larynx 2008;35:579–82. 18201851 10.1016/j.anl.2007.10.009

[16] Li L Zhou F Lin F. Clinicopathologic characteristics of thyroid-like low-grade nasopharyngeal papillary adenocarcinoma: a case report. Appl Immunohistochem Mol Morphol 2019;27:e81–4. 29494399 10.1097/PAI.0000000000000545

